# Bacterial Community Diversity of Oil-Contaminated Soils Assessed by High Throughput Sequencing of 16S rRNA Genes

**DOI:** 10.3390/ijerph121012002

**Published:** 2015-09-24

**Authors:** Mu Peng, Xiaoxue Zi, Qiuyu Wang

**Affiliations:** College of Life Science, Northeast Forestry University, No.26 Hexing Street, Xiangfang District, Harbin 150040, China; E-Mails: huangxiaoming321@sina.com (M.P.); L19813211@163.com (X.Z.)

**Keywords:** oil-contaminated soils, 454 Pyrosequencing, 16S rRNA, bacterial community, bacterial diversity

## Abstract

Soil bacteria play a major role in ecological and biodegradable function processes in oil-contaminated soils. Here, we assessed the bacterial diversity and changes therein in oil-contaminated soils exposed to different periods of oil pollution using 454 pyrosequencing of 16S rRNA genes. No less than 24,953 valid reads and 6246 operational taxonomic units (OTUs) were obtained from all five studied samples. OTU richness was relatively higher in contaminated soils than clean samples. *Acidobacteria*, *Actinobacteria*, *Bacteroidetes*, *Chloroflexi*, *Planctomycetes* and *Proteobacteria* were the dominant phyla among all the soil samples. The heatmap plot depicted the relative percentage of each bacterial family within each sample and clustered five samples into two groups. For the samples, bacteria in the soils varied at different periods of oil exposure. The oil pollution exerted strong selective pressure to propagate many potentially petroleum degrading bacteria. Redundancy analysis (RDA) indicated that organic matter was the highest determinant factor for explaining the variations in community compositions. This suggests that compared to clean soils, oil-polluted soils support more diverse bacterial communities and soil bacterial community shifts were mainly controlled by organic matter and exposure time. These results provide some useful information for bioremediation of petroleum contaminated soil in the future.

## 1. Introduction

Crude oil is a complex mixture of hydrocarbons and other organic compounds that can bring up serious environmental problems when spills occur [1,2]. Despite the economic benefits of the crude oil industry, oil spillage influences and alters soil microbial community composition and biogeochemical cycles, and triggers strongly negative impacts in sustainable soil fertility and environmental quality. The petroleum pollutants entered into soil influence plant growth and cause human health concerns [3].

Soil is a privileged habitat for microorganisms and is the most biodiverse environment on Earth [4]. Soil microorganisms are a very important part of the environmental ecosystems, which could adjust energy flow and cycle of matter by digesting animal, plant and oil residues, and play a pivotal role in growth and development of agricultural crops, balance of the soil ecosystem, organic matter transfer and bioremediation. Furthermore, the diversity of the microbial community in soil is closely related to the function and structure of its surrounding ecosystem, and is one of the components to maintain soil productivity. The present research in this field is mainly focused on species diversity [5], genetic diversity [6], structural diversity [7] and functional diversity [8].

Molecular methods have greatly helped us understand the microbial community and diversity [9]. However, for complex environmental samples, such as wastewater and soil with overwhelming genetic diversities, these methods are still far away from disclosing the panorama of the bacterial community and can only detect the most abundant population in the samples [10,11]. Next generation sequencing techniques could overcome this shortage and limitation. Pyrosequencing of 16S rRNA genes, developed by Roche 454 Life Science using sequencing-by-synthesis technology, is one of the popular high throughout sequencing systems and a powerful approach for investigating the bacterial communities in the environment [12,13]. Recently, this technology has been successfully used in analyzing microbial communities and diversity in various environmental samples, such as wastewater [10], biosolids [12], and oil-contaminated soil [14].

Characterization of bacterial communities living in oil-contaminated soils and evaluation of their oil-degradation capacities could potentially serve as guide for bioremediation of the polluted environments. Therefore, in this study, we investigated the bacterial structure and diversity in the oil-contaminated soils exposed to different times of oil pollution using the pyrosequencing approach. The purpose is to help us better understand the dynamics of bacterial communities in oil-contaminated soils and provide useful and appropriate information for bioremediation strategies of petroleum contaminated soil in the future.

## 2. Materials and Methods

### 2.1. Study Site and Sample Treatment

The experimental site was situated in the Daqing oilfield at 45°46′ to 46°55′ north between 124°19′ and 125°12′ longitude, and the elevation in 640 m, which is an important oil producing area in China and the world. This area has the continental monsoon climate where the annual rainfall ranges from 400 mm to 550 mm and the mean annual temperature is 4.2 °C [15]. The local soil type is saline-alkaline.

In this study, the five soil samples were collected from one district. Two of them close to the abandoned wells mined in 1960s (JBT60) and 1970s (JBT70) respectively, where soil samples polluted by oil during exploration activities in 1960s and 1970s, were collected around the well within 50 meters. One soil (SYT) was directly sampled from a recent (one year) oil spilled pit with residual oil, the last two samples (JBT1 and JBT2) were collected from fresh saline-alkali soil surrounding the oil-contaminated sampling site as controls, with the basic characteristics of the soil shown in Table 1.

Soil samples were collected at depths from the surface down to 20 cm by the five-point sampling method in three plots of each sampling site, a part of which were then pooled and homogenized within the same sterilized plastic bag for detecting soil bacteria diversity. The other part was used for detecting the basic soil properties. The soil water content was measured by drying 10 g sieved soil at 105 °C for 8 h and soil pH was measured in the suspension of soil and water using a 1:2.5 ratio, electric conductivity (EC) of soil leaching solution was determined using a hand-held electrical conductivity meter (FiveEasy30, Mettler Toledo, Sweden) [16], with each treatment in three replications.

### 2.2. DNA Extraction and PCR Amplification of Bacterial 16S rRNA Gene

Total genomic DNA was extracted from 1 g the soil samples using E.Z.N.A. Soil DNA Kit (OMEGA, Georgia, GA, USA) following a modified protocol to the manufacture’s protocols. The DNA extracted in each sampling for three times and pooled together, then stored in −20 °C for downstream manipulation. Bacterial 16S rRNA genes were amplified using the following universal primers added a 10-nucleotide barcode: 27F (5′-AGAGTTTGATCCTGGCTCAG-3′) and 533R (5′-TTACCGCGGCTGCTGGCAC-3′) [17]. PCR reaction was performed in 20 μL volumes containing 10 ng of template DNA, 5 μM primers (0.8 μL), 2.5 mM each dNTP (2 μL), 4 μL 5 × FastPfu Buffer, and FastPfu Polymerase (0.4 μL) (Applied Biosystems, California, CA, USA). The DNA amplification was performed under the following cycling conditions: 1 cycle of 2 min at 94 °C, then 25 cycles of 30 s at 94 °C, 30 s at 55 °C and 1 min at 72 °C, followed by a final cycle of 10 min at 72 °C. Then the products were checked by 2% agarose gel to confirm 450 bp DNA amplicons. 

### 2.3. High Throughput Sequencing of Bacterial 16S rRNA Gene

PCR products were purified by a PCR Purification Kit (OMEGA, USA). The 200 ng purified products used for 454 pyrosequencing on the Roche 454 GS-FLX Titanium sequencer (Roche 454 Life Sciences, Branford, CT, USA) at Majorbio Bio Technology Co. Ltd., Shanghai, China. 

### 2.4. Statistical and Bioinformatics Analysis

The data of soil basic properties was analyzed with the Statistical Product and Service Solutions 17.0 (SPSS 17.0, SPSS Inc., Chicago, CA, USA) software package. To improve the quality of pyrosequencing data and eliminate the effect of random sequencing errors, we deleted some defective data from the libraries, including an average q-value below 25, singleton, reads shorter than 200 bp, the forward primer and barcode of each read, and those containing ambiguous base call [18]. These pyrosequencing reads were clustered into operational taxonomic units (OTUs) (sequences that have 97% similarity were defined as one OTU) [19]. Then the OTUs were further clustered using the MOTHUR program with sequence distances set at 0.03. Based on these clusters, the Alpha-diversity parameters were calculated for each sample: Chao1 estimator, ACE estimator, abundance-based coverage estimator, Shannon and Simpson index, and the rarefaction curve at 0.03 using Sigmaplot 11.0 software (Systat Software Inc., California, CA, USA). A principal component analysis (PCA) was carried out based on weighted UniFrac distance. BLAST of taxonomic classification down to the phylum, class and genus level was carried out using Ribosomal Database Project with a set confidence threshold of 80%. In addition, heatmap, Venn diagram and Redundancy analysis (RDA) were drawn by hierarchal clustering performed according to Gentleman and Ihaka [20].

## 3. Results

### 3.1. Physicochemical Properties of Soil

Seven physicochemical characteristics of the soil samples were shown in Table 1. All soils were saline-alkali with a pH above 7. Three oil-contaminated soils (JBT60, JBT70, SYT) showed a gradient of pH levels, ranging from 7.36 to 8.05. Two clean soils (JBT1, JBT2) had comparable concentrations of pH with the highest value. The two clean soils harbored the highest water content and EC values among all samples (Table 1), meaning that some basic soil properties might have been changed by oil contamination and soil microbial activity. Total nitrogen, total phosphorus and organic matter were significantly different in oil-contaminated soils exposed to different times of oil pollution. The results showed the significant differences in all parameters between oil-contaminated samples and control samples.

### 3.2. Microbial Richness and Diversity

A total of 24,953 valid reads were obtained from the five samples by 454 pyrosequencing analysis (Table 2), with number of sequences ranging from 2132 (SYT) to 8187 (JBT2) at 3% distance. Among all soil samples, a total of 6246 different OTUs were revealed with the highest value in JBT60 (1917 OTUs) and lowest in JBT1 (852 OTUs), however, this difference could be attributed to the variation in sequencing depth and the limited number of reads. The rarefaction curves, especially for the JBT60 and SYT samples, did not reach the saturation plateau, indicating that new bacterial phylotypes would still be valuable to be detected even after 8000 reads detected by pyrosequencing (Figure S1).

### 3.3. Taxonomic Composition

**Figure 1:**
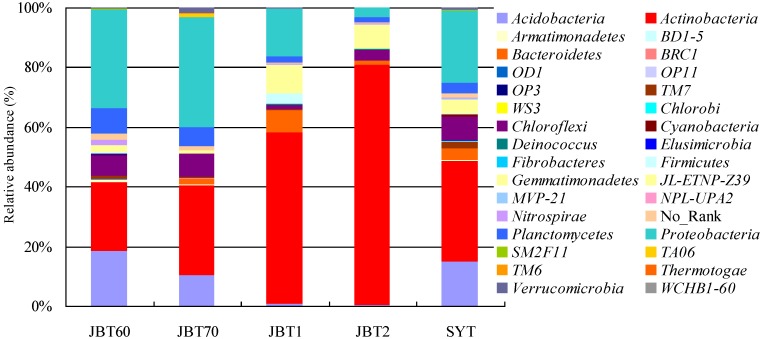
Relative abundance of predominant bacterial composition in the five samples. The abbreviation of samples is shown in Table 1.

Twenty-nine different phyla were observed from these sites, in which *Acidobacteria*, *Actinobacteria*, *Bacteroidetes*, *Chloroflexi*, *Planctomycetes* and *Proteobacteria* were the dominant phyla, and accounted for over 80% of the reads in each sample (Figure S2 and Table S1). However, the bacterial community abundance of phyla was significantly difference in the five samples (Figure 1). The most abundant OTUs associated with the JBT60 library were the sequences related to *Acidobacteria*, *Actinobacteria*, and *Proteobacteria*, whereas the same trend was found in JBT70 with different account. The most abundance sequences in the JBT1 library were those related to *Actinobacteria*, *Bacteroidetes* and *Proteobacteria*. The JBT2 library was largely dominated by sequences related to *Actinobacteria*, while the SYT library, was numerically dominated in the sequences related to *Acidobacteria*, *Actinobacteria*, *Chloroflexi*, and *Proteobacteria* (Figure 1).

**Table 1:** The basic properties of the soil samples used in this study.

Parameters	JBT60	JBT70	JBT1	SYT	JBT2
Soil characteristics	The oil contaminated soil from abandoned well mined in 1960s	The oil contaminated soil from abandoned well mined in 1970	The Control closest to JBT60/70	The oil contaminated soil from recent (one year) oil spilled site	The Control closest to SYT
pH	7.36 ± 0.11 dD	8.05 ± 0.04 bB	9.40 ± 0.03 aA	7.90 ± 0.05 cC	9.35 ± 0.01 aA
Soil water content (%)	7.37 ± 0.39 cC	5.65 ± 0.30 dD	14.17 ± 0.41 aA	9.09 ± 0.29 bB	13.52 ± 0.46 aA
EC (uS/cm)	75.60 ± 3.06 eE	192.30 ± 2.10 dD	1716.43 ± 16.82 aA	578.36 ± 6.15 cC	1225.17 ± 10.41 bB
Total N (mg/g)	1.30 ± 0.02 aA	0.55 ± 0.03 cC	0.70 ± 0.09 bB	0.55 ± 0.01 cC	0.70 ± 0.04 bB
Total P (mg/g)	0.22 ± 0.01 aA	0.32 ± 0.01 bB	0.19 ± 0.01 bB	0.21 ± 0.02 bB	0.31 ± 0.05 aA
Organic content (%)	1.86 ± 0.31 dC	6.01 ± 0.31 bB	5.24 ± 0.13 cB	18.69 ± 0.69 aA	5.34 ± 0.01 cB

Note: The data in the same column used Duncan’s new multiple range method to count for the mean and standard deviation. Lowercase and capital letters stand for significant difference at 5% and 1% level, respectively.

**Table 2:** Similarity-based OTUs and species richness estimates of the bacterial phylotypes in the five samples. The abbreviation of samples is shown in Table 1.

Sample ID	Valid Reads	Cluster Distance (0.03)					
		OTU	ACE	Chao1	Coverage	Shannon	Simpson
JBT60	4097	1917	8338	4832	0.68	6.98	0.002
JBT70	5777	1320	3582	2602	0.88	5.92	0.012
JBT1	4760	852	2437	1663	0.90	5.11	0.026
SYT	2132	1161	4217	2838	0.63	6.70	0.0017
JBT2	8187	996	2437	1799	0.94	5.37	0.012
Total	24953	6246	--	--	--	--	--

--, Not Available.

### 3.4. Relationships between Bacterial Communities among the Five Samples

**Figure 2:**
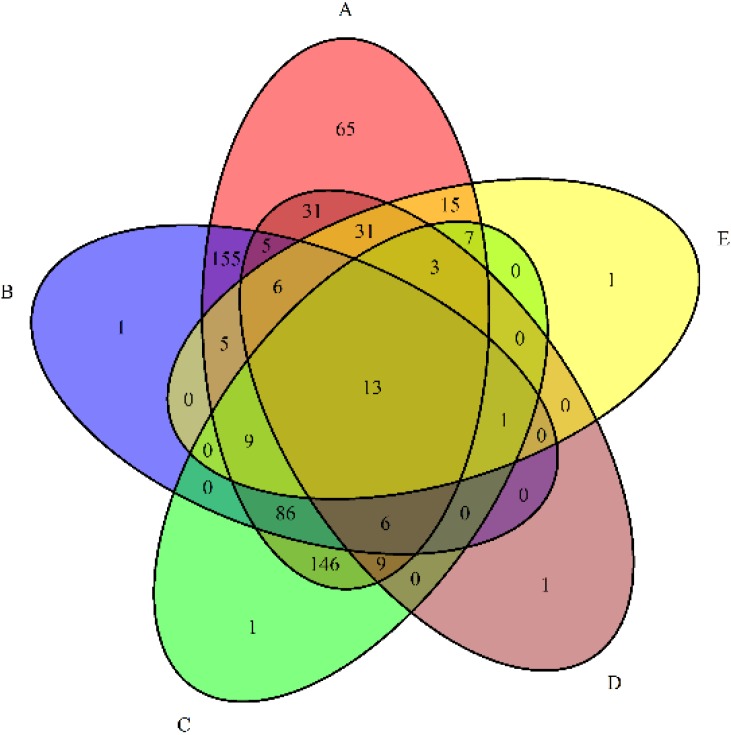
Venn diagram denoting the number of unique and shared species in the different libraries at 3% distance level. **A**: JBT60 sample, **B**: JBT70 sample, **C**: JBT1 sample, **D**: JBT2 sample, **E**: SYT sample. The abbreviation of samples is shown in Table 1.

**Figure 3:**
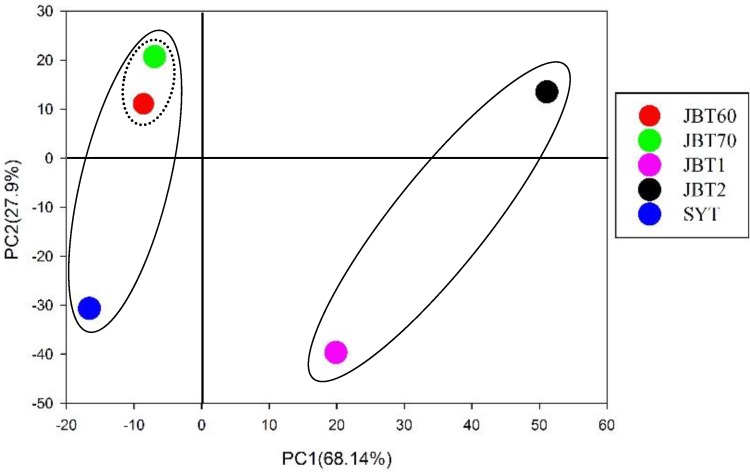
The principal component analysis of samples at the genus level. Principal component 1 and 2 explained 68.14% and 27.9% of the total variations, respectively. Based on PC1, JBT1/2 were clustered together, JBT60/70 and SYT were in the same group (solid lines). JBT60/70 and SYT were further divided into two groups (dashed line). The abbreviation of samples is shown in Table 1.

A venn diagram was analyzed to find the common species among these libraries [21]. The results showed that the number of species shared among all libraries was 13 (Figure 2). The SYT library shared 90 and 34 bacterial species with contaminated samples (JBT60 and JBT70), while 26 and 54 common species were shared among SYT with control soils. The principal component analysis based on weighted UniFrac distance and heatmap analysis in relation to soil basic characteristics and bacterial community properties by pyrosequencing data were performed to reveal the relationships among the five samples at the genus level [22]. The principal component analysis score plot (Figure 3) depicted that JBT1 and JBT2 grouped to the right of the graph along the first principal component (PC1), while JBT60/70 and SYT were closely related on the left of PC1, which represented 68.14% of the total variations. The JBT60 and JBT70 samples fell into a cluster, whereas the SYT sample exhibited a noticeable and regular separation from JBT60/70 at the upside of the graph along the second principal component (PC2), which accounted for 27.9% of the total variations, indicating an entirely different community structure (Figure 3). Overall, both of the principal components were involved in 96.04% of the total variations of the different communities, and could explain most variations of the sample bacteria.

**Figure 4:**
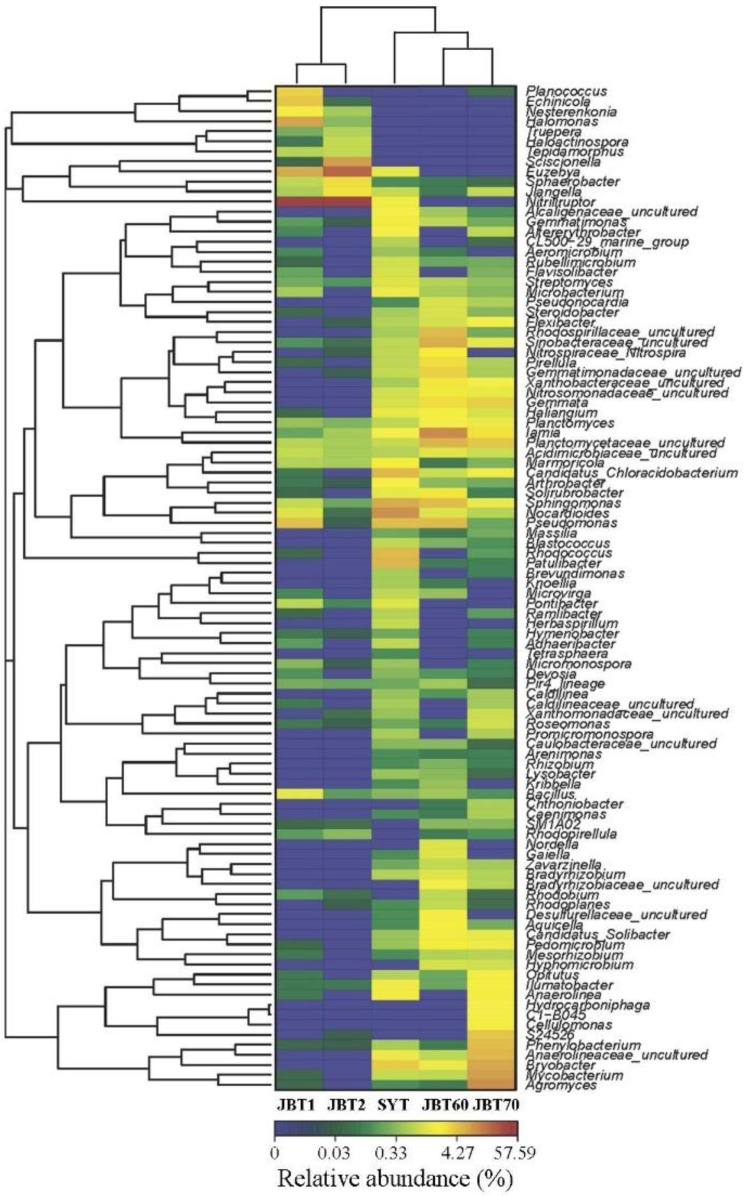
Hierarchically clustered heatmap of bacterial distribution of different communities from the five samples at the genus level. Row represents the relative percentage of each bacterial genus, and column stands for different samples. The relative abundance for each bacterial genus were depicted by color intensity with the legend indicated at the under of the figure. The abbreviation of samples is shown in Table 1.

To get an overall view on the identified connections among the studied samples, a hierarchically clustered heatmap was performed. The heatmap plot depicted the relative percentage of each bacterial family (variables clustering on the Y-axis) within each sample (X-axis clustering) (Figure 4). As shown in Figure 4, JBT60 and JBT70 libraries grouped firstly together, and then they clustered with SYT. Finally, these two groups gathered by decreasing order of similarity. The relative abundance for each bacterial genus was depicted by color intensity with the legend indicated in the figure below. In this study, the majority of sequences belonged to *Euzebya* (4.66%–12.26%) and *Nitriliruptor* (39.45%–32.64%) were present in the two clean soils, whereas it was absent in long-term polluted soils. Sequence belonging to *Chloracidobacterium*, *Arthrobacter*, *Nocardioides*, *Sphingomonas* and *Nocardioides* were the most dominant bacterial genuses in the SYT sample but were rarely in the other soils. The bacterial genus *Agromyces*, *Mycobacterium*, *Bryobacter*, *Anaerolineaceae*, *Phenylobacterium* and *Cellulomonas* were only found in the JBT70 with relatively higher abundance but was rarely or not detected in the others. While in JBY60 sample, the dominant genera were distributed among *Gemmatimonadaceae*, *Pirellula*, *Nitrospiraceae*, *Lamia*, and *Planctomycetaceae*. The proteobacterial sequences belonged to different genera in the three oil-contaminated soils, with no common genera. The majority of *Proteobacteria* belonged to the genus *Rhodospirillaceae*, *Sinobacteraceae* in JBT60 but belonged to *Hydrocarboniphaga* and *Pseudomonas*, *Rhodococcus* in JBT70 and SYT, respectively. The remaining abundant genera *Streptomyces*, *Bacillus*, *Marmoricola*, *llumatobacter* were detected in all samples. From the distribution of color, blue was mainly found in two control samples, indicating that oil-contaminated samples were enriched and highly diverse when to compare with the control samples. The results of the PCA and heatmap analyses were in agreement. Both the analysis indicated that the oil-contaminated soils and control samples had different characteristic bacterial communities.

**Figure 5:**
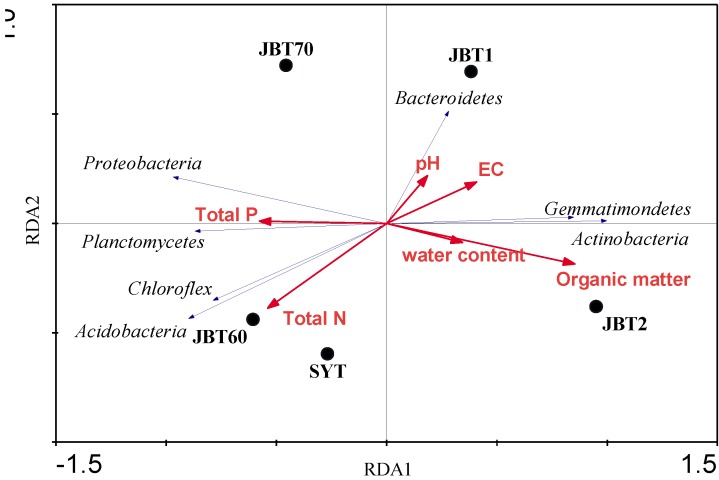
Redundancy analysis (RDA) of the relationship between the soil physicochemical parameters and the relative abundance of bacterial phylum of the five soil samples (*P* < 0.05). Solid circles represent the soil sample. The abbreviation of samples is shown in Table 1. Arrows indicated the direction and magnitude of variables.

Redundancy analysis (RDA) indicated that organic matter was the highest determinant factor for explaining the most variations in the community compositions of the five soil samples (Figure 5). While the JBT1 sample had high pH and electric conductivity (EC) values and low total P, the soil SYT and JBT60 were related with relatively lower levels of pH and EC but higher total P and N. The relative abundance of some phyla, *Gemmatimonadetes* and *Actinobacteria* were correlated with increasing organic matter concentrations, while *Bacteroidetes* were related with increasing pH and EC. The bacterial phyla, *Planctomycetes*, *Acidobacteria* and *Chloroflex* were apparently most dominant in JBT60 and SYT soils.

## 4. Discussions

To date, extensive research has focused on oil bioremediation using pure cultures or mixed bacterial consortia isolated from oil spilled soils. However, only few studies have been reported on the different bacterial communities and diversity in soils, which were contaminated at different times of oil exposure [14,23]. Here, we analyzed the bacterial diversity in oil-contaminated soils, and the OTUs, species richness estimates and heatmap results showed that higher bacterial diversity in polluted soils than that of control samples in the Daqing oilfield (Table 2 and Figure 4). 

The results of Sutton, *et al.* [24] on the microbial community composition and diversity at 26 samples in the long-term diesel-contaminated soil showed that *Proteobacteria*, *Firmicutes*, *Actinobacteria*, *Acidobacteria* and *Chloroflexi* were the major microbial groups. However, as shown in our work (Figure 1), the top six phyla were *Acidobacteria*, *Actinobacteria*, *Bacteroidetes*, *Chloroflexi*, *Planctomycetes* and *Proteobacteria*, among which *Actinobacteria* and *Planctomycetales* are the environmental-friendly bacteria used for degrading contaminants. The high abundance of *Acidimicrobiales* found in all samples was mainly due to the saline-alkali soil of the collected sites, which also was agreement with pH value (Table 1). Therefore, the dominant bacterial groups in our study were significantly different from those of Sutton *et al.* [24] and Dos Santos *et al.* [14] (Tables S1–S3). However, some low abundance groups, *Elusimicrobia* and *Deinococcus*, were found in our study, but not detected by other methods in relevant work [25].

As shown in a hierarchically clustered heatmap of bacterial distribution (Figure 4), it is interesting to find that many dominant species detected in the contaminated samples were related to oil-degrading strains, such as *Acinetobacter*, *Bacillus*, *Nocardia*, *Solirubrobacter*, and *Sphingomonas*, which corresponds with previous reports. Ruberto, *et al.* [26] reported that the hydrocarbon-degrading strain B-2-2, that belongs to the genus *Acinetobacter*, was the dominant population of soil polluted by oil or petrol in Antarctic. *Actinobacteria* were the most versatile and efficient culturable hydrocarbon degraders [27]. Chan [28] isolated dominant bacteria (*Bacillus* sp.), demonstrated to function in the biodegradation in petroleum oil. And a report further evidenced that *Bacillus* has the ability to degrade petroleum hydrocarbon and aromatic compounds, such as chlorophenol, which were mostly found in pollution soil [2]. Zhang *et al.* [1] collected the oil-contaminated soils from the Daqing oilfield and isolated 19 bacterial strains related to *Bacillus* species, which could be used for bioremediation or biotreatment of oil-polluted soils. In another report, *Nocardia* was shown to degrade the total petroleum hydrocarbons [29].

Although few studies could isolate *Solirubrobacter* from the natural environment, we detected this special bacteria in the SYT sample with a high relative abundance, and suggest that it might participate in the oil degradation process. In addition, *Sphingomonas* isolated from the sterile and contaminated soils were found with polycyclic aromatic hydrocarbons-degrading ability [30]. Species of *Pseudomonas* have been reported to be the most prevailing group of bacteria to degrade complex organic compounds, including polycyclic aromatic hydrocarbon (PAHs) [31], biphenyl [32] and other aromatic contaminants [33]. In the present study, relative abundance analysis found *Solirubrobacter*, *Sphingomonas*, *Nocardioides* and *Pseudomonas* to be the most dominant in JBT60, JBT70 and SYT, and at low abundance in in JBT1 and JBT2, clearly indicating that oil polluted soil contains unique oil-degrading bacterial communities. Interestingly the occurrence of the genera *Anaerolinea* was novel in our oil-contaminated soil samples (JBT70 and SYT), basically separated from a thermophilic sludge, and largely involved in utilizing *N*-acetyl-glucosamine [34]. In addition, an interesting feature of the sequences belonging to *Nitriliruptor* is found in JBT1 and JBT2 with the highest abundance (32.64%–39.45%) in this study, indicating that these bacteria widely exist in saline-alkali soil and might be associated with salinity adaptability, which is consistent with Sorokin *et al*. [35].

Sutton *et al.* [24] clearly underscored that the presence of oil contamination significantly influences bacterial community structure and diversity, regardless of the soil matrix type, and suggested that clean samples had higher diversity than contaminated soil. However, compared with their research, we found an opposite result that was the control soils (clean samples) had lower diversity than polluted soil (Table 2 and Figure 4). The reason might be that our control samples were from the saline-alkali soil where it is difficult for many bacteria to survive, except for some particular bacterial groups. In addition, oil pollution may selectively stimulate some bacterial propagation for petroleum degradation, which is a regular abundant member in the contaminated soil adapted for the biodegradation of pollutants (Figure 3). Interestingly, RDA analysis showed a significant influence of organic matter level on the bacterial diversity and distribution between the clean and contaminated samples (Figure 5), while other previous studies indicated that pH values were the important drivers of bacterial community structure [36,37]. In this survey, only 2.04 pH units existed and the significant differences in soil EC and organic matter were observed in different soil samples.

A further important and interesting phenomenon found here is that some bacterial community compositions only existed in the three contaminated samples under the different times of oil exposure (Figure 3). This suggests that the bacterial community composition may change over time [38]. For example, *Acidobacteriaceae*, *Acidothermus*, *Desulfurellaceae*, *etc.* were only found in JBT60, while *Actinoplanes*, *Anaerolinea*, *Caldilinea*, *etc.* were only detected in JBT70. In addition, *Adhaeribacter*, *Herbaspirillum*, *Promicromonospora*, *etc*. were only detected in SYT (Figure 3). *Phenylobacterium*, which efficiently degrades linear alkylbenzene sulphonic acid, was found in both JBT70 and SYT with high relative abundance. This is an indication that each step of the oil degradation process has its special and unique bacterial groups; similar findings were reported in Dos Santos *et al.* [14]. Furthermore, a significant difference of the bacterial species was found in JBT60, JBT70 and SYT samples, each included 592, 287 and 91 specific species, respectively, demonstrating that samples with long-term oil exposure had higher bacterial diversity. And some bacteria were described a primary role in the degradation of aromatic hydrocarbons released in a contaminated environment [39], which could justify bacterial predominance after oil contamination and assist in the removal of contaminant. These interesting results were also consistent with Ke *et al.* [38] and Dos Santos *et al.* [14]. 

This study reported soil bacterial diversity variation induced by oil pollution. Soil bacterial community composition and diversity were characterized using barcoded pyrosequencing. The results show that, compared to clean soils, oil-polluted soils supported more diverse bacterial communities. Soil bacterial community shifts were mainly controlled by exposure time in oil. This study provides data to help support our understanding for bioremediation of petroleum-contaminated soil using oil-degrading bacteria.

## Supplementary Files

Supplementary File 1
