# Supplementary material for: Bacterial Community Diversity of Oil-Contaminated Soils Assessed by High Throughput Sequencing of 16S rRNA Genes
Source: Int J Environ Res Public Health. 2015 Sep 24;12(10):12002–15. doi: 10.3390/ijerph121012002 (PMC4626951; doi:10.3390/ijerph121012002)
Supplement: Supplementary File 1 [file ijerph-12-12002-s001.pdf]

Supplementary Information

Table S1. Relative abundances of bacterial phyla in the studied soils. Values represent percentages of all sequences assigned to the bacteria for soils. The description of sample name is shown in Table 1.

Phylum	JBT60	JBT70	SYT	JBT1	JBT2
Acidobacteria	18.53	10.14	14.73	0.67	0.38
Actinobacteria	22.92	30.14	33.77	57.52	80.62
Armatimonadetes	0.63	0.33	0.61	0.04	0
candidate division BD1-5	0.02	0.03	0	0	0
Bacteroidetes	0.51	1.96	3.61	7.5	1.4
candidate division BRC1	0.07	0.24	0.09	0.02	0.04
candidate division OD1	0.2	0.02	0	0.02	0
candidate division OP11	0.05	0	0	0	0
candidate division OP3	0.1	0	0	0	0
candidate division TM7	0.44	0.36	2.02	0.06	0
candidate division WS3	0.05	0	0.05	0	0
Chlorobi	0.12	0.05	0.28	0	0
Chloroflexi	6.83	7.67	8.49	1.55	3.37
Cyanobacteria	0.24	0.1	0.47	0.02	0
Deinococcus	0	0	0	0.17	0.37
Elusimicrobia	0.22	0	0.05	0	0
Fibrobacteres	0.12	0.02	0	0	0
Firmicutes	0.22	0.16	0.33	3.7	0.09
Gemmatimonadetes	2.61	0.8	4.64	9.6	8.02
candidate division JL-ETNP-Z39	0.02	0	0	0	0
candidate division MVP-21	0.02	0	0.38	0	0
Nitrospirae	1.76	0.02	0.42	0	0
Planctomycetes	8.42	6.58	3.52	2.04	1.69
Proteobacteria	33.27	36.71	24.2	16.26	3.3
candidate division SM2F11	0.05	0.02	0.14	0	0
candidate division TA06	0	1.16	0.05	0.02	0
candidate division TM6	0.07	0.14	0	0	0
Verrucomicrobia	0.44	1.89	0.75	0	0
candidate division WCHB1-60	0	0.02	0	0.19	0

Table S2. Relative abundances of bacterial class in the studied soils. Values represent percentages of all sequences assigned to the bacteria for soils. The description of sample name is shown in Table 1.

Class	JBT60	JBT70	SYT	JBT1	JBT2
Acidimicrobiia	12.06	8.71	6.14	2.71	3.76
Acidobacteria	16.82	7.56	11.26	0.59	0.21
Actinobacteria	3.44	16.31	18.34	8.68	20.61
Alphaproteobacteria	16.94	15.15	13.32	6.01	2.71
Anaerolineae	0.44	3.72	2.16	0.04	0
Armatimonadia	0	0.05	0.09	0.02	0
Bacilli	0.17	0.09	0.23	3.66	0.09
Bacteroidia	0	0.36	0	0.06	0
Betaproteobacteria	2.66	4.22	3.75	0.17	0.02
Caldilineae	0.05	0.45	0.42	0.04	0
Chlorobia	0.12	0.03	0.23	0	0
Chloroflexi	0.15	0.14	0.47	0.02	0
Chthonomonadetes	0	0.09	0	0	0
Clostridia	0.05	0.07	0.09	0.04	0
Coriobacteriia	0.02	0.02	0	0	0
Cyanobacteria(no_rank)	0	0	0.33	0.02	0
Cytophagia	0.37	1.04	1.59	6.72	1.39
Deinococci	0	0	0	0.17	0.37
Deltaproteobacteria	5.61	2.63	3.05	0.76	0.24
Elusimicrobia	0.22	0	0.05	0	0
Fibrobacteria	0.12	0.02	0	0	0
Flavobacteria	0	0.02	0	0.42	0
Gammaproteobacteria	7.13	14.61	4.03	9.31	0.32
Gemmatimonadetes	2.61	0.8	4.64	9.6	8.02
Holophagae	1.27	2.58	3.47	0.08	0.17
Ktedonobacteria	0.02	0	0.09	0	0
Nitriliruptoria	0	0	1.41	44.16	45.17
Nitrospira	1.76	0.02	0.42	0.46	0.44
No_Rank	7.03	4	5.39	0.86	1.92
Opitutae	0.1	0.9	0.28	0.08	0
Phycisphaerae	1.34	0.76	1.22	0.4	0.46
Planctomycetacia	6.42	5.54	2.25	1.64	1.22
Rubrobacteria	0.12	0.09	0.28	0	0
Spartobacteria	0.12	0.61	0.19	0.02	0
Sphaerobacteridae{subclass}	0.02	0.02	0.05	0.5	1.82
Sphingobacteriia	0.15	0.52	1.88	0.17	0.01
TakashiAC-B11	0.12	0	0.09	0.17	0.32
Thermoleophilia	4.69	4.81	6.43	1.05	8.4
Thermomicrobia	0.1	0.29	0.75	0.11	0.7
Thermotogae	0.07	0.12	0	0	0
Verrucomicrobiae	12.06	8.71	6.14	0.02	0

Table S3. Relative abundances of bacterial order in the studied soils. Values represent percentages of all sequences assigned to the bacteria for soils. The description of sample name is shown in Table 1.

Order	JBT60	JBT70	JBT1	JBT2	SYT
Acidimicrobiales	12.06	8.71	2.71	3.76	6.14
Acidithiobacillales	0.81	8.53	0.00	0.00	0.00
Acidobacteria_Order_Incertae_Sedis	0.59	3.31	0.00	0.00	1.88
Acidobacteriales	0.63	0.00	0.00	0.00	0.09
Alteromonadales	0.00	0.99	0.02	0.00	0.00
Anaerolineales	0.44	3.72	0.04	0.00	2.16
Armatimonadales	0.00	0.05	0.02	0.00	0.09
Bacillales	0.17	0.09	3.66	0.09	0.23
Bacteroidales	0.00	0.36	0.06	0.00	0.00
Bdellovibrionales	0.17	0.00	0.00	0.00	0.00
Burkholderiales	0.34	2.70	0.17	0.02	2.63
Caldilineales	0.05	0.45	0.04	0.00	0.42
Caulobacterales	0.39	2.44	0.02	0.02	0.75
Chlorobiales	0.12	0.03	0.00	0.00	0.23
Chloroflexales	0.10	0.09	0.00	0.00	0.19
Chromatiales	0.00	0.00	0.00	0.00	0.05
Chthoniobacterales	0.12	0.61	0.02	0.00	0.19
Chthonomonadales	0.00	0.09	0.00	0.00	0.00
Clostridiales	0.05	0.07	0.04	0.00	0.09
Coriobacteriales	0.02	0.02	0.00	0.00	0.00
Corynebacteriales	0.34	4.00	0.71	0.06	3.56
Cytophagales	0.37	1.02	4.01	0.13	1.41
Deinococcales	0.00	0.00	0.17	0.37	0.00
Desulfobacterales	0.00	0.50	0.00	0.00	0.14
Desulfurellales	0.68	0.00	0.00	0.00	0.05
Desulfuromonadales	0.02	0.23	0.00	0.01	0.14
Enterobacteriales	0.00	0.05	0.00	0.00	0.05
Euzebyales	0.00	0.00	4.66	12.26	0.61
Fibrobacterales	0.10	0.02	0.00	0.00	0.00
Flavobacteriales	0.00	0.02	0.42	0.00	0.00
Frankiales	0.17	0.33	0.27	0.31	2.16
Gaiellales	1.07	0.19	0.23	0.37	0.75
Gammaproteobacteria_Order_Incertae_Sedis	0.00	0.00	0.17	0.01	0.00
Gemmatimonadales	2.12	0.43	0.29	0.09	2.53
Glycomycetales	0.00	0.00	0.02	0.02	0.00
Hydrogenophilales	0.00	0.40	0.00	0.00	0.00
Ignavibacteriales	0.00	0.02	0.00	0.00	0.05
Kineosporiales	0.02	0.00	0.02	0.00	0.19
Legionellales	0.76	0.24	0.13	0.00	0.14
Micrococcales	0.46	9.54	2.48	0.43	3.33
Micromonosporales	0.29	0.19	0.29	0.07	0.28
Myxococcales	1.93	1.82	0.04	0.01	1.22

Table S3. *Cont.*

Order	JBT60	JBT70	JBT1	JBT2	SYT
Nitriliruptorales	0.00	0.00	39.45	32.64	0.80
Nitrosomonadales	1.00	0.64	0.00	0.00	0.42
Nitrospirales	1.76	0.02	0.46	0.44	0.42
Oceanospirillales	0.00	0.00	5.27	0.18	0.00
Opitutales	0.10	0.88	0.04	0.00	0.28
Phycisphaerales	0.27	0.17	0.23	0.32	0.00
Planctomycetales	6.42	5.54	1.64	1.22	2.25
Propionibacteriales	0.66	1.61	1.68	2.04	7.04
Pseudomonadales	2.00	0.09	3.36	0.01	2.30
Pseudonocardiales	0.59	0.45	0.04	4.76	0.56
Puniceicoccales	0.00	0.02	0.02	0.00	0.00
Rhizobiales	7.13	5.5	3.74	1.22	3.00
Rhodobacterales	0.10	0.36	0.59	0.09	0.56
Rhodocyclales	0.00	0.07	0.00	0.00	0.33
Rhodospirillales	6.22	4.22	0.82	0.87	1.41
Rickettsiales	0.07	0.05	0.04	0.05	0.19
Rubrobacterales	0.12	0.09	0.00	0.00	0.28
Solirubrobacterales	3.61	4.62	0.80	8.02	5.63
Sphaerobacterales	0.02	0.02	0.50	1.82	0.05
Sphingobacteriales	0.15	0.52	0.17	0.01	1.88
Sphingomonadales	2.68	1.52	0.78	0.42	7.27
Streptomycetales	0.24	0.17	0.15	0.07	0.52
Streptosporangiales	0.02	0.00	0.08	0.77	0.00
Syntrophobacterales	0.07	0.05	0.00	0.00	0.00
Verrucomicrobiales	0.07	0.12	0.02	0.00	0.00
Xanthomonadales	3.37	2.73	0.11	0.06	1.27

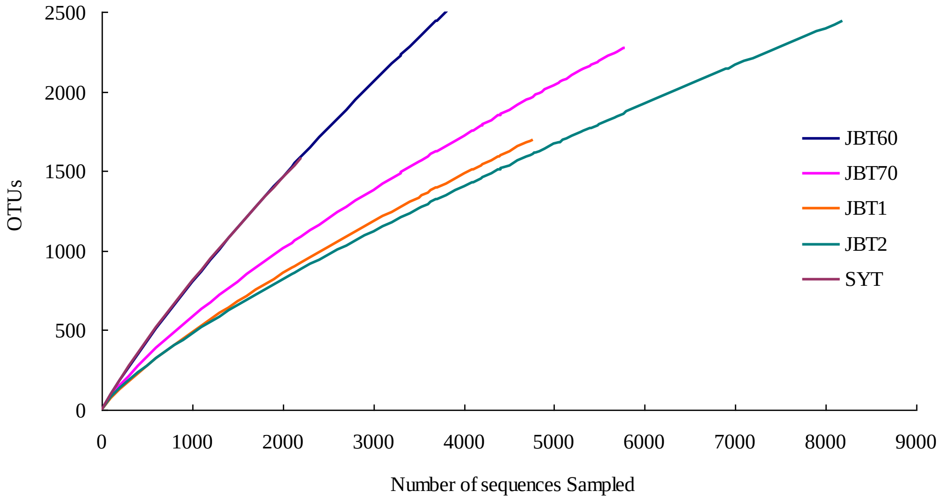

Figure S1. Rarefaction curves based on 16S rRNA sequences among the different samples. The curves were generated for 97% levels of OTUs. The abbreviation of samples is shown in Table 1.

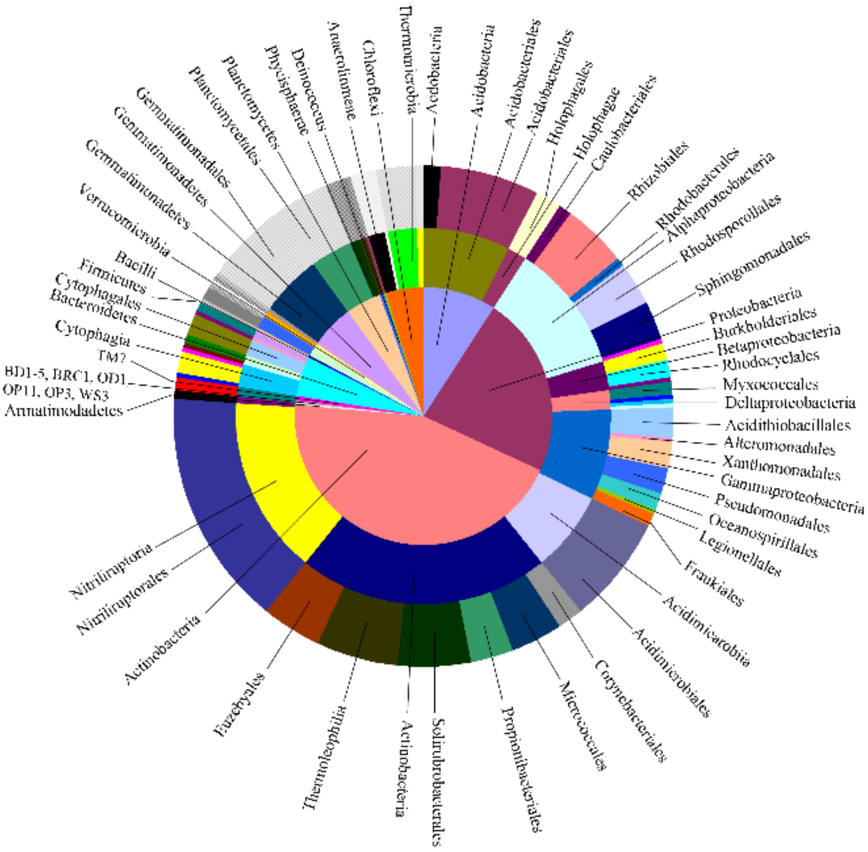

Figure S2. Bacterial diversity richness and phylogenetic distribution on phylum (**Inner Circle**), class (**Middle Circle**), and order level (**Outer Circle**).

© 2015 by the authors; licensee MDPI, Basel, Switzerland. This article is an open access article distributed under the terms and conditions of the Creative Commons Attribution license (http://creativecommons.org/licenses/by/4.0/).
